# Dual Targeting of the Insulin-Like Growth Factor and Collateral Pathways in Cancer: Combating Drug Resistance

**DOI:** 10.3390/cancers3033029

**Published:** 2011-07-26

**Authors:** Joseph A. Ludwig, Salah-Eddine Lamhamedi-Cherradi, Ho-Young Lee, Aung Naing, Robert Benjamin

**Affiliations:** 1 Departments of Sarcoma Medical Oncology, The University of Texas MD Anderson Cancer Center, Houston, Texas 77030, USA; E-Mails: slamhamedi@mdanderson.org (S.L.C.); rbenjami@mdanderson.org (R.B.); 2 Departments of Thoracic Head & Neck Medical Oncology, The University of Texas MD Anderson Cancer Center, Houston, Texas 77030, USA; E-Mail: hlee@mdanderson.org (H.Y.L.); 3 Investigational Cancer Therapeutics, The University of Texas MD Anderson Cancer Center, Houston, Texas 77030, USA; E-Mail: anaing@mdanderson.org (A.N.)

**Keywords:** IGF-1R, insulin-like growth factor, combination therapy, drug resistance, Ewing sarcoma

## Abstract

The insulin-like growth factor pathway, regulated by a complex interplay of growth factors, cognate receptors, and binding proteins, is critically important for many of the hallmarks of cancer such as oncogenesis, cell division, growth, and antineoplastic resistance. Naturally, a number of clinical trials have sought to directly abrogate insulin-like growth factor receptor 1 (IGF-1R) function and/or indirectly mitigate its downstream mediators such as mTOR, PI3K, MAPK, and others under the assumption that such therapeutic interventions would provide clinical benefit, demonstrable by impaired tumor growth as well as prolonged progression-free and overall survival for patients. Though a small subset of patients enrolled within phase I or II clinical trials revealed dramatic clinical response to IGF-1R targeted therapies (most using monoclonal antibodies to IGF-1R), in toto, the anticancer effect has been underwhelming and unsustained, as even those with marked clinical responses seem to rapidly acquire resistance to IGF-1R targeted agents when used alone through yet to be identified mechanisms. As the IGF-1R receptor is just one of many that converge upon common intracellular signaling cascades, it is likely that effective IGF-1R targeting must occur in parallel with blockade of redundant signaling paths. Herein, we present the rationale for dual targeting of IGF-1R and other signaling molecules as an effective strategy to combat acquired drug resistance by carcinomas and sarcomas.

## 1. Introduction

Since 1957, IGF-1 and IGF-2 (historically referred to as somatomedins A and C, respectively) were identified as second messengers of growth hormone (GH) capable of promoting insulin-like anabolic effects upon normal somatic tissues such as skeletal muscle and bone [1]. Since then, they and their cognate receptors have been demonstrated to affect a diverse range of cancers by facilitating malignant transformation, altering cell differentiation, and promoting cancer growth, metastasis, and chemotherapy resistance. However, only within the last decade have physicians had at their disposal both a host of clinically relevant anti-IGF-1R targeted therapies (small molecules and antibody-based inhibitors against IGF-1R or IGF-1) and a number of diverse high throughput technology platforms capable of readily teasing apart the multifaceted pharmacodynamic effects exerted by such IGF-1R antagonism.

Whilst a number of excellent reviews have thoroughly discussed the impact of IGF-1R stimulation upon normal and malignant cells [2-7], or highlighted the myriad therapeutic options under preclinical and clinical investigation, few have concisely narrowed the focus to the complex interplay that exists between IGF-1R and a host of redundant signaling pathways (e.g., integrins, her2/neu, EGFR) potentially responsible for both de novo and acquired resistance to IGF-1R and its downstream targets. Following a necessarily brief summary of the IGF-1R family and its cancer promoting effects, the crux of this review has been dedicated to mechanisms of IGF-1R resistance and dual-targeted strategies aimed at circumventing them. We conclude by drawing upon the success of other targeted therapies (such as trastuzumab or imatinib) and suggest a rational path forward in IGF-1R centric trial design that integrates pharmacodynamic biomarkers to improve patient selection for likely responders and enhance our monitoring of the molecular changes induced by IGF-1R targeting.

## 2. IGF-1R Receptor Family

### 2.1. IR and IGF-1R Receptors

Three receptors (Insulin receptor, IGF-1R and IGF-2R), their respective ligands (insulin, IGF-1, and IGF-2), and six IGF binding proteins (IGFBP1-6) comprise a phylogenetically well-conserved signaling family that strongly influences anabolic and metabolic control over both physiologic and aberrant cellular processes. First identified, and more closely linked to metabolism, the insulin receptor (IR) is a hetero-tetrameric receptor tyrosine kinase (RTK) composed of two identical heterodimers forged from α- and β-chains linked by covalent disulfide bonding. Two isoforms exist; isoform A, which lacks the twelve amino-acids coded by exon 11 within the carboxy-terminus of the α-subunit (residues 717-729), or Isoform B that contains the replete amino-acid sequence [8]. A close structural analog of the IR, IGF-1R has nearly 61% overall homology to the insulin receptor, is 84% homologous at the kinase domains, and nearly identical within the ATP-binding pocket [9-11]. The IGF-2R lacks a functional intracellular β-chain but may inversely affect IGF-1R signaling by sequestering IGF-2, a less potent IGF-1R binding partner.

For both the IR and IGF-1R, cleavage of the pro-receptor spawns a 130-kDa α-chain and a 90-kDa β-chain. They are joined within the cytoplasm, glycosylated, and folded under the direction of calnexin and calreticulin (both chaperones) then, as ‘half-receptors’, migrate to the plasma membrane where they float freely, able to interact with themselves or, rarely, with other RTK. Adding a layer of complexity to the IGF-1R pathway, the α- and β-isoforms of IR may associate with the IGF-1R half-receptor to form functionally active hybrid receptors (hybrid-R), mainly when IR and IGF-1R are heavily expressed or imbalanced in their expression, as would be expected to occur following sequential internalization and down-regulation induced by a number of IGF-1R-targeted therapeutic antibodies now undergoing clinical investigation. Under these circumstances, such hybrid receptors can become the predominant vehicle by which IGF-1, IGF-2, and, to lesser extent insulin, exert their oncogenic effects. As will be discussed later, though most drug candidates targeting the IGF-1R pathway were selected for their ability to preferentially inhibit IGF-1R rather than IR (out of concern regarding the perceived and sometimes real risk of hyperglycemia), less stringent binding to also include the hybrid receptors is increasingly considered of potential value.

X-ray crystallography has solved the three-dimensional structure of both IR [12] and IGF-1R [13,14] in both their unphosphorylated (inactive) and phosphorylated states. The ligand-binding α-domains of IR and IGF-1R are purely extracellular, whereas the β-domain includes extracellular, transmembrane, and intracellular segments – the latter is comprised of a kinase domain and two regulatory regions. The more proximal juxta-membrane regulatory region serves as a docking site for adaptor proteins such as IRS-1 and SHC, whereas a distal one includes an activation loop capable of acting as an autoinhibitory pseudosubstrate that blocks the tyrosine-binding site in the inactive, closed confirmation until trans-phosphorylation occurs. The exact mechanism of ligand-induced trans-phosphorylation of the adjoined α-β IGF-1R heterodimer is unclear, but is thought to occur through receptor oligomerization or altered conformational change of pre-coupled heterodimers [15,16].

### 2.2. Ligands and IGF-Binding Proteins

As opposed to physiological levels of insulin (0.5 nmol/L), secreted by the pancreatic β-cells, circulating IGF-1 (20 nmol/L) and IGF-2 (90 nmol/L) can be produced by the liver (under the influence of growth hormone), by malignant tissues themselves, or by their associated stroma; thus, they may stimulate cancers through endocrine, autocrine, or paracrine effects, respectively [17-20]. IGF-2 is frequently over-expressed by normal and cancerous tissues due to a loss of imprinting. Modulating ligand bioactivity, the six IGFBPs generally act to intercede with ligand-receptor binding. Whereas IGFBP3 provides the greatest IGF-1 binding and serves to prolong its serum half-life, IGFBP2 and IGFBP5 can rarely have the opposite effect, possibly by enhancing ligand distribution and release into neoplastic tissues. Though early attempts to suppress circulating IGF-1 (using hormonal agents such as octreotide or IGFBP-mimetics) failed, nascent approaches have been more promising.

### 2.3. Downstream Signaling

Unlike other tyrosine kinase receptors, like her2/neu for example, gene amplification and/or mutations in the IGF-1R gene are distinctly uncommon, occurring in less than ten percent of breast cancers [21] and rarely in pediatric wild-type gastrointestinal stromal tumors [22], pancreatic adenocarcinomas [23], and Wilms' tumors [24]. Constitutive activation of the IGF-1R receptor also appears infrequently [25]. Instead, enhanced IGF-1R signaling generally occurs through greater ligand binding and/or increased IGF-1R expression followed by secondary propagation through two principal pathways, the mitogen-activation protein kinase pathway (MAPK) and the phosphatidylinositol-3-kinase (PI3-K)/Akt/mTOR pathway (extensively reviewed elsewhere) [26-29].

**Figure 1.**
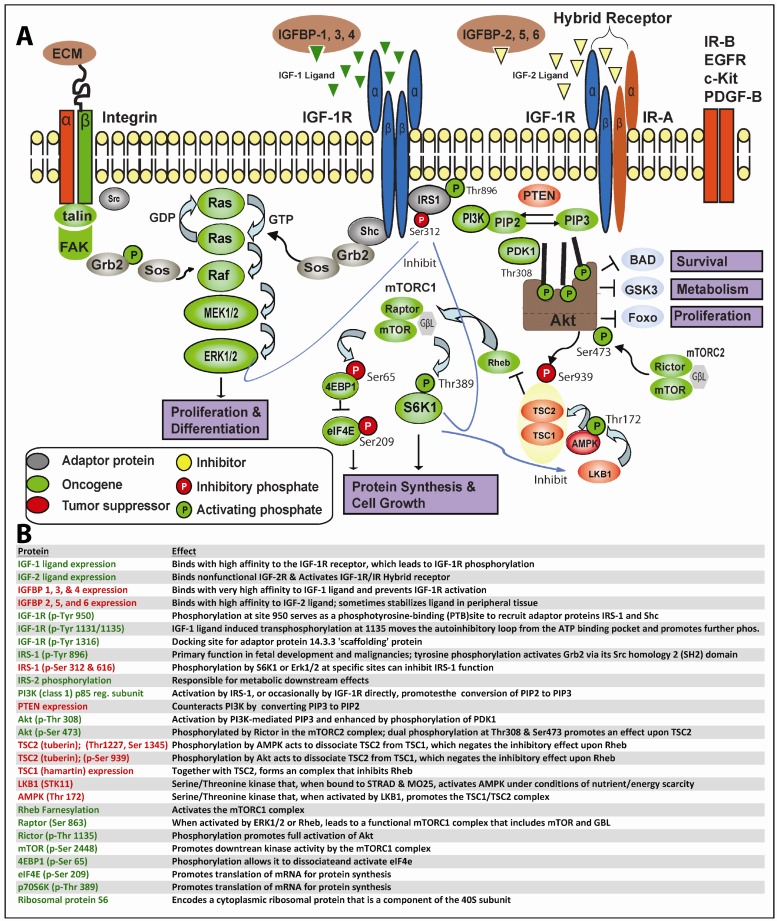
Key proteins and phosphorylation states necessary for IGF-1R signaling, shown schematically (A) and with full description of each protein's potential effect (B). Although multiple downstream pathways exist, several including MAPK and Akt/mTOR are critical for cell proliferation, differentiation, protein synthesis, cell survival, and metabolism.

In greater depth, as shown in Figure 1, this process begins when IGF-1 or IGF-2 (albeit with reduced affinity) binds to IGF-1R or the IGF-1R/IR hybrid receptors, resulting in trans-phosphorylation of the IGF-1R tyrosine kinase domain and other critical sites. Secondary phosphorylation of IGF-1R at amino acid 950 results in enhanced interaction with the phosphotyrosine binding (PTB) site of IRS-1 principally, but also IRS-2, which themselves becomes tyrosine-phosphorylated; IRS-1/2 may occasionally become constitutively phosphorylated independent of IGF-1R [30]. Adaptor proteins (such as Shc, Grb2, CrkII, CrkL, Sos) may bind to one of eighteen unique Src homology 2 (SH2) or PTB binding sites on IRS-1 and, thereby, stimulate the sequential activation of Ras, Raf, MEK1/2 and ERK1/2 along the MAPK cascade. Alternatively, IGF-1R-mediated activation of IRS-1/2 can recruit PI3K to the plasma membrane, where it catalyzes the conversion from PIP2 to PIP3. PIP3 in turn activates 3-phosphoinositide-dependant protein kinase 1 (PDK1) and downstream Akt. Finally, Akt, through inhibition of tuberous sclerosis complex 2 (TSC2), which regulates Rheb, mTOR becomes activated. A recent report indicates PDK1 can be tyrosine phosphorylated by direct IGF-1R binding [31]. As is readily apparent, one implication of the lengthy and often tortuous nonlinear path from proximal IGF-1R signaling to final activation of MAPK and/or mTOR is that neoplastic cells have ample means to maintain downstream IGF-1R signaling despite well-intentioned IGF-1R targeting.

## 3. IGF-1R and Cancer: Pathway Signaling & Single-agent Targeted Therapy

Given the capacity of IGF-1R to initiate both normal and pathological signaling cascades, IGF-1R and its downstream mediators have, naturally, been widely implicated in contributing to various malignancies and been investigated as potential therapeutic targets now for more than two decades. High IGF-1 levels have been found in several sarcoma subtypes [32,33] and IGF-1R overexpression in breast, lung, prostate, or colon cancer has been shown to accelerate cancer progression [34-37] and enable anchorage-independent growth [17]. Conversely, congenital syndromes resulting in IGF-1 or growth hormone deficiency likely confer protective effects [38,39].

The inner workings of the IGF-1R molecular machinery share considerable similarities between carcinomas and sarcomas, both from a myopic view at the level of biologically conserved protein-protein interactions that can be understood with reasonable specificity, and from the higher vantage point of a ‘signaling network’ that can be anticipated to progress, if not deterministically, at least under stochastic rules gleaned from years of scientific scrutiny. Yet, considerable biologically complexity exists not only from one cancer type to the next but also, as is often the case, among asynchronously responding tumors within the same patient.

In addition to the intrinsic differences present within the IGF-1 pathway itself (via altered expression of IGF-1R, its ligand, the IGFBPs, or downstream effectors, for example), such changes only partially explain the varied sensitivity to IGF-1 oriented therapies. Extrinsic differences independent of the IGF-1R pathway, such as her2/neu or EGFR activation for breast or lung cancer, respectively, likely play a larger role since those pathways often serve as the primary regulator of their malignant phenotype. Under that scenario, the IGF-1 pathway would theoretically be redundant, and largely quiescent, until the primary pathway becomes impaired, either spontaneously or secondary to therapeutic intervention. Ultimately, then, the importance of IGF-1 signaling can vary tremendously across cancer types and even temporally within a patient's tumor if selective pressure is applied through therapeutic targeting.

### 3.1. Carcinomas

Among carcinomas, IGF-1R targeting has been most widely evaluated in breast, lung, colon, and pancreatic cancer and numerous reviews have outlined its impact, or absence thereof, in detail elsewhere. Therefore, only limited information is summarized below to illustrate how dual targeted therapy could likely prove beneficial, given the multifaceted interaction between the IGF-1R pathway and others that converge upon MAPK or mTOR.

#### 3.1.1. Breast Cancer

High IGF-1R expression has been observed in breast cancer cell lines and human tissue specimens, leading to increased activity of this pathway [40]. Just as hybrid IGF-1R/IR receptors may occur, IGF-1R may pair with ErbB2 or EGFR to form functional hybrid receptors capable of enhancing downstream mediators of survival and proliferation (cyclins, E2F4, survivin, HIF1a, Bcl2, Bax, c-Myc, *etc.*) [27,41,42]. IGF-1R over-expression has been associated with resistance to trastuzumab and pertuzumab therapies *in vitro* [43-45] and its inhibition, via IGF-1R targeted small interfering RNA (siRNA) or tyrosine kinase inhibitors (like NVP-AEW541) appears to counteract those resistance mechanisms [46,47]. Thus, there is a strong rational for dual targeting of IGF-1R and ErbB2 or EGFR in breast or other carcinomas such as lung cancer [48].

#### 3.1.2. Prostate Cancer

Like breast cancer, prostate cancers are often controlled by several regulatory effects of growth factors such as IGF-1, EGF, FGF-β, and KGF [49]. Antibody-mediated inhibition of IGF-1R can result in significant inhibition of tumor growth in both androgen independent and dependent xenograft models [50,51] and IGF-1R/EGFR crosstalk has been associated with resistance to gefitinib in the well-characterized DU145 prostate cancer cell line. Numerous studies have shown that dual inhibition of IGF-1R and EGFR can be synergistic in moderating growth and migration of prostate cancer [43], lung cancer [52-54], and colorectal cancer [55,56].

#### 3.1.3. Colon Cancer

Expression of IGF-1R by immunohistochemistry has been found in more than half of colon cancer specimens [57] or cell lines [58], and its affect upon colon cancer oncogenesis and progression has been observed in a number of preclinical models [59]. However, the clinical effects of IGF-1R signaling remain less certain. Whereas a prospective study by Ma *et al.* associated high levels of IGF-1, and low IGFBP3, with an increased risk of colon cancer, this same group later reported no associated link between IGF-1 and patients at high risk of acquiring colon cancer [60,61]. Furthermore, among patients treated for nonmetastatic colorectal cancer, neither IGF-1 nor IGFBP3 expression affected mortality rates. Finally, in a randomized phase II trial of IMC-A12 used alone or in combination with cetuximab in patients with advanced colorectal cancer, none of the twenty-three patients treated with the single-agent IGF-1R antibody responded [62]. Therefore, IGF-1R directed therapy likely offers little if any benefit for this cancer type.

#### 3.1.4. Pancreatic Cancer

The IGF-1R signaling cascade has been implicated in the development and progression of pancreatic cancer [63-66], and naturally, both preclinical [25,67-70] and clinical studies directed at this pathway have been initiated. For example, one clinical trial that targets EGFR and IGF-1R in combination with gemcitabine (NCT00617708) has recently closed, while a similar trial at our institution (using a different IGF-1R directed antibody) is still ongoing (NCT00769483). Another, assessing a single-agent IGF-1R antibody in local unresectable or metastatic pancreatic neuroendocrine tumors remains open as well (NCT01024387). Though it is too early to judge of the efficacy of IGF-1R therapies, alone or in combination with other targeted agents in pancreatic cancer, preliminary results suggest they're relatively well tolerated even in patients with advanced cancer stage.

#### 3.1.5. Lung Cancer

Deregulation of IGF signaling has been described in both non-small cell lung cancer (NSCLC) and SCLC [71,72]. Furthermore, elevated plasma levels of IGF-1 have been associated with an increased risk of lung cancer and high plasma levels of IGFBP3 have been associated with a reduced risk, although results from a meta-analysis did not recapitulate this association [73-75]. Among several IGF-1R single-agent antibodies in various stages of clinical development, figitumumab has been tested most extensively [76,77], however, recent phase III trials of figitumumab were terminated due to an apparent imbalance of serious adverse events and excess mortality in the experimental arm. As might be expected, there are several ongoing clinical trials combining IGF-1R targeted antibodies with traditional cytotoxics or EGFR inhibitors such as erlotinib in NSCLC patients. A phase II randomized trial, called IMPACT, is recruiting patients affected by advanced non-squamous NSCLC to receive cisplatin plus pemetrexed with or without weekly MK-0646 as first-line therapy [78].

As we have learned from EGFR inhibitors, the challenge for IGF-1R inhibitors will consequently correspond to how we optimally select patients who could benefit most from these agents. Interestingly, IGF-1R overexpression has been shown in squamous cell carcinoma and recent studies suggested that it could serve as a predictive biomarker of response to anti-IGF-1R antibody, such as R1507[54] or figitumumab [79]. Based on a quantitative immunohistochemical analysis of patients sample from the phase II trial of figitumumab [77], the epithelial-mesenchymal transition (EMT) status of cancer might be a candidate biomarker of response rate to the combination of chemotherapy and figitumumab [79], which needs further elucidation. Although these studies were not designed to investigate the activity according to NSCLC histologies or EMT status, these results suggest rational strategy to enrich for lung cancer patients that might benefit from treatment with anti-IGF-1R antibodies. Furthermore, EGFR and K-ras mutations have been implicated as biomarkers for selecting patients in IGF-1R TKI-based therapy for NSCLC patients (Kim WY *et al.*, *AACR 2010 Annual Meeting*, Abstract # 4127). In this study, introduction of mutant K-Ras induced IGF-1R TKI resistance, while a knockout of mutant K-Ras restored the sensitivity in *in vitro* and *in vivo* models. These findings emphasize the need to produce more robust preclinical, early clinical and translational data to be successful in larger randomized trials.

### 3.2. Sarcomas

Since more than fifty sarcomas subtypes exist, each clinically and molecularly distinct from one another and often driven by unique pathognomonic genomic translocations, the effects of IGF-1R signaling are naturally varied and subtype specific. Hirschfeld and Helman first described a role for IGF-1R in tumor promotion of pediatric solid tumors including osteosarcoma and Ewing's sarcoma (EWS), and since then, preclinical studies have confirmed that IGF-1R autocrine signaling is important for not only their pathogenesis but for a number of soft-tissue sarcomas as well [32].

With respect to EWS, the major focus of our laboratory, the ubiquitously expressed IGF-1R receptor works in concert with the most common EWS oncogenic fusion protein (EWS-FLI-1) to promote tumorigenesis. IGF-1R activation is required for EWS-FLI-1 induced malignant transformation of murine fibroblasts [80] and, when transfected in mouse progenitor cells, the EWS-FLI-1 fusion protein (but not native FLI-1 or ERG) is reported to induce a nearly nine-fold increase in IGF-1 expression – directly linking the most common EWS translocation to IGF-1 autocrine signaling [81]. Furthermore, upon binding of the EWS/FLI-1 fusion protein to the insulin like growth factor binding protein (IGFBP-3) promoter, IGFBP-3 transcriptional activity is reduced, free IGFBP-3 decreases, and more IGF-I ligand is available for ligand-induced activation of the ubiquitous IGF-1R [10,82,83]. Thus, autocrine loops may occur both through up-regulation of total IGF-1 and increased availability for IGF-1R binding. A final auto-stimulatory circuit occurs via up-regulation of IGF-1R itself, as is the case of desmoplastic small round cell tumors (DSCRT), an even rarer sarcoma subtype the bares substantial molecular and clinical similarities to EWS. For DSRCT, the EWS-WT1 fusion protein has been reported to increase IGF-1R promoter activity by 3.4-fold.

Although a mouse monoclonal antibody (αIR-3) was first shown to be effective against *in vivo* rhabdomyosarcoma in 1986, translation to the clinic was slow, secondary to human-anti mouse Ab formation. Small molecule targeting of IGF-1R had different challenges, related to the close homology between IGF-1R and IR in the TK domains. Using a class of fully humanized anti-IGF-1R Ab, made possible through recombinant technologies, unexpectedly high clinical response rates for sarcoma subtypes (specifically EWS and osteosarcoma) have renewed academic interests in IGF-1R targeted therapies and, consequently, nearly a dozen phase I/II trials are currently underway evaluating IGF-1R targeted monoclonal antibodies (mAb) or small molecules for the treatment of EWS [84,85]. Preclinical evidence suggests that IGF-1R signaling is likely to be vital for soft-tissue sarcomas as well. As stated previously, although *IGF1R* mutations are distinctly uncommon in tumors, genetic polymorphisms exist in genes that encode IGF-1 and IGFBP-3 [27]. Elevated IGF-1R expression has been linked to *IGF1R* amplification, which infrequently occurs in wild-type (WT) gastrointestinal stromal tumors (GIST) that lack prototypical gain-of-function c-kit receptor mutations [22], but over-expression is the norm in pediatric WT GISTs even in the absence of such amplification events [86]. It's still too early to predict from ongoing clinical trials which sarcoma subtypes will ultimately benefit the most from IGF-1R targeting, as unexpected clinical responses have occurred in several diverse subtypes (e.g., solitary fibrous tumors, liposarcoma, and others).

In preclinical animal models using antibodies to IGF-1R, no antibody dependent cellular toxicity (ADCC) or cross-reactivity to the insulin receptor has been observed. Though still presented only in abstract form at ASCO, the preliminary results of the SARC-11 trial (a multicenter, open-label, multi-arm, phase II study of R1507 for the treatment of patients with recurrent or metastatic, drug-refractory EWS and selected other sarcomas) were disappointing; this trial, and two smaller ones, exhibited treatment responses less than 9%. Interestingly, nearly one-third of patients exhibited early treatment response after 9–14 days (as assessed by PET/CT in several trials) but the vast majority of such responders rapidly progressed thereafter, presumably due to acquired resistance. Although in most cases the early imaging findings were not used as metrics of clinical response, they nevertheless point to a much higher, albeit brief, signal of activity that could prove clinically meaningful if acquired resistance mechanisms can be identified and thwarted.

For a description of binding specificities for IGF-1R, IR, and/or hybrid receptors [87] and comprehensive review of the dozen or more IGF-1R-targeted single-agent antibodies or small molecules now in preclinical development or early phase clinical trials, one may refer to a number of excellent reviews highlighting their possible therapeutic value for cancer in general [2,3,7,20,88-90], and sarcoma [6,33,91] or carcinoma [5,59,92] in particular.

## 4. Resistance Mechanisms to IGF-1R-targeted Therapy

Notwithstanding a clear benefit observed in a small subset of patients treated with single-agent IGF-1R antagonists, enthusiasm for single-agent IGF-1R targeting has waned and most active or developing clinical trials have evolved to use IGF-1R-targeted therapies together with others that surmount anticipated mechanisms of resistance. Of course the resistance mechanisms themselves have only partially been enumerated and, as discussed previously, they likely vary from one cancer type to another, subject to the predominant oncogenic driver (Table 1).

Putative mechanisms of resistance may be conceptually grouped by two broad categories:

1. Primary independence from IGF-1R activation, presumably through myriad pathways that bypass IGF-1R (*i.e.*, upstream plasma membrane bound receptors including alternative RTKs and hybrid receptor combinations that also activate Grb2, Sos, or IRS-1) or downstream molecules capable of intrinsic self-activation of MAPK and Akt/mTOR.
2. Direct counterregulatory effects within the IGF-1R system, including up-regulated expression or phosphorylation of IGF-1R, increased expression or availability of ligands, and altered modulation by IGFBPs.

With respect to the first category or resistance, cross talk via alternative RTK or non-receptor transmembrane signalers (such as integrins) could potentially bypass the need for IGF-1R signaling. In addition to EGFR, PDGF-β [93], NGF-R [10], and HER2 expression [94], some sarcomas have been shown to express c-kit [93,94]. Imatinib-induced shutdown of c-kit receptor phosphorylation leads to a 20–30% reduction in EWS cell proliferation and suppressed tumor growth in xenograft models, albeit at doses 20-fold higher than that used for treatment of gastrointestinal stromal tumors (18–22 μM) [93,95]. Used alone, less than 5% of EWS patients achieve a partial response to single-agent imatinib (440 mg/m^2^/day) [96]. Dasatinib, a multi-targeted tyrosine kinase inhibitor (TKI) of c-kit and PDGF-β has also shown activity, again at high concentrations [97]. Given the partial overlap IGF-1R antagonists and of the c-kit or PDGF-β TKIs (which predominately suppress MAPK), one may hypothesize that c-kit or PDGF-β up-regulation is a potential mechanism of IGF-1R resistance. The synergy observed *in vitro* between small molecule antagonists of the IGF-1R (such as NVP-ADW742 or NVP-AEW541) and imatinib, through apoptotic mechanisms, supports this hypothesis although, to our knowledge, secondary up-regulation of those receptors in IGF-1R-resistant cells has yet to be shown [98].

Other receptors, including the epidermal growth factor receptor (EGFR), the vascular endothelial growth factor receptor-2 (VEGFR-2), and rearranged in transformation (RET) kinase receptor have been evaluated and another, macrophage-stimulating 1 receptor tyrosine kinase (MST1R) has just recently been identified as potential means to induce IGF-1R-independent stimulation [99,100]. Though gefitinib (an EGFR kinase inhibitor) and vandetanib (an inhibitor of VEGFR-2, VEGFR-3, and RET kinase) inhibited EWS growth at high concentrations (greater than 5 μM), nonspecific effects were suspected since the phosphorylation state of MAPK and Akt were unchanged. Scotlandi *et al.* has reported HER2 expression in 16% of EWS specimens, however gene amplification was absent and little antiproliferative response to trastuzumab (Herceptin) was observed [94]. In summary, of the experience of non-IGF-1R tyrosine kinase inhibitors for EWS treatment, none has significant single-agent activity in the setting of functional IGF-1R. This does not, of course, rule out their role in IGF-1R-resistant tumors; the additive and/or synergistic effects reported in combination with either of the Novartis's pyrrolo[2,3-d]pyrimidine derivatives or Bristol Myers Squibb's pyrrolecarboxaldehydes (BMS-554417 or BMS-536924), in fact, suggests compensatory signaling could occur under IGF-1R-null conditions, as has been recently reported by Helman [101]. Adding a layer of complexity, since insulin and IGF-1 half-receptors have been reported to form heterodimers with members of the EGFR family in lung cancer, this adds another layer of complexity in assessing TKI-mediated resistance [72].

Regarding the second category of IGF-1R resistance, complex counterregulatory loops in the IGF-1R autocrine circuit, including the receptors, ligands, and binding-proteins, may be involved. One such example is the autoregulatory loop between Mdm2 and p53. Froment *et al.* have reported that Mdm2, a protein antagonist of p53, can physically bind IGF-1R and target it for ubiquitination-induced degradation independent of p53 [102,103]. Interestingly, whereas wild type-p53 down-regulates transcription of IGF-1R at the promoter level, mutant p53 induced the opposite effect in osteosarcoma and rhabdomyosarcoma cells. Since p53 mutations are observed in less than 5% of EWS primary tumors [104], it remains to be determined whether mutant-p53-induced up-regulation of IGF-1R exists as a resistance mechanism for IGF-1R targeted therapy.

The level or activation status of members of the IGF-1R family may affect resistance. Since neither mutation nor amplification is common, this is not the most likely contributor to antibody resistance. Though not yet confirmed to be prognostic in EWS, high levels of IGF-1R appear to confer sensitivity in rhabdomyosarcoma, and may serve as a valid prognostic biomarker for that cancer [105]. Low levels of IGF-1R may, conversely, confer resistance in at least two ways: (1) IGF-1R-low-expressing cells would theoretically be less reliant or ‘addicted’ upon IGF-1R for growth and; (2) targeted therapies generally requires a paired target for effectiveness [106]. Paradoxically, high IGF-1R levels, when stabilized by Heat Shock Protein-90 (HSP90; a chaperone protein that helps maintain stability, renature unfolded proteins, or targeted their degradation), may also confer at least short-term resistance as hypothesized by Martins *et al.* [95]. In evaluating why HSP-90 was transiently elevated in ADW742-resistant A673 EWS cells, it was suggested that client-protein stabilization of activated IGF-1R or and Akt by HSP90, maintained downstream signaling of the Akt/mTOR pathway.

In the most recent and comprehensive report of IGF-1R resistance mechanisms to use genetic and proteomic profiling, Helman compared BMS-536924-resistant sarcoma and neuroblastoma cells to sensitive ones, thereby identifying gene and protein subsets that significantly correlated with *de novo* drug sensitivity. Although members of the IGF-1R family did not reach statistical significance for *a priori* inclusion within those subgroups, high IGF-1R, IGF-1, or IGF-2 levels portended sensitivity whereas elevated IGFBPs 3 and 6 were higher in resistant cell lines. Unexpectedly, the combination of IGF-1 and IGF-2 into a single model was better than either used alone in predicting response, suggesting an active role for both ligands in IGF-1R signaling. While an IGF-2-mediated effect may not be intuitive, since IGF-2 has twenty-fold less affinity for IGF-1R, it has recently been reported that malignancies can shift their reliance from the paradigmatic IGF-1-stimulated IGF-1R pathway instead to an IGF-2-stimulated one that acts upon the IGF-1R/IR-α hybrid receptor already mentioned [107].

As suggested earlier, given the capacity for tumor-associated stoma to secrete IGF-1 or IGF-2, paracrine loops may also affect the efficacy of IGF-1R targeted therapy. Gorlick, Houghton, and others have reported relative insensitivity to IGF-1R- or mTOR-targeted therapies *in vitro* compared to xenografts models of similar tumor types, supporting our hypothesis that extracellular mechanisms of resistance are important [108]. Since tumor regrowth (after initial response) is a near universal occurrence in xenograft models (reportedly with continued IGF-1R downregulation and maintained p-Akt) [108] and patients treated with single-agent IGF-1R targeted therapies to date, a fresh approach must seek to obviate not only IGF-1R signaling but also the cancer type-specific resistance mechanism(s) as well.

Although many of the IGF-1R resistance mechanisms described above pertain to sarcomas, major mechanisms of resistance can be found across the spectrum of diverse cancer types. At other times, the mechanism(s) of resistance are unique and specific to the individual features that distinguish one cancer from another, as identified for the most common cancer types within Table 1.

## 5. Combating IGF-1R Resistance: Dual Targeting and Beyond

Even before the precise mechanisms of single-agent IGF-1R success, and in some cases failure, are thoroughly scrutinized, a limited number of preclinical studies and mostly early phase clinical trials have begun to assess the safety and efficacy of dual targeting of IGF-1R and putative secondary targets suspected of enabling acquired IGF-1R resistance (Table 2). Paralleling the defined mechanisms of resistance highlighted above, multi-targeted therapy can target key components intrinsic to the IGF-1R receptor family (the receptors, ligands, or IGFBPs in various combinations) or extrinsic ones.

An example of intrinsic targeting includes MEDI-573, a dual IGF-1/2 targeted neutralizing antibody that can theoretically prevent activation of both IGF-1R and IGF-1R/IR-A hybrid receptors. Similar in effect, small molecule inhibitors of IR and IGF-1R, such as OSI-906, have generated significant enthusiasm, given anecdotal reports of clinical response [109-111]. Though not in clinical trials, yet another approach combines two antibodies that together target divergent epitopes within the ligand binding sites of IGF-1R [112]. Each of those therapeutic strategies offer to inhibit IGF-1R function while countering the compensatory IR-mediated crosstalk inherent in IR-A and its pairing with IGF-1R [88]. Whereas both IGF-1 and IGF-2 ligands bind and activate IGF-1R and IGF-1R/IR-A hybrid receptors, the additional suppression of IGF-2 may limit unopposed IR-A signaling [113]. Given the increased expression IR-A within neoplastic tissues and preferential affect upon IGF-induced mitogenic signaling, as opposed to IR-B that exerts greater influence upon glucose hemostasis within normal liver, muscle and fat, one could conceivably target just IGF-1R and the oncogenic IR-A splice variant while minimizing hyperglycemia and untoward side effects associated with down-regulated IR-B. In practice, however, this hypothesis remains to be proven and, to our knowledge, there are no selective IR-A inhibitors. Because elevated levels of plasma IGF-1 and insulin occur as respective feedback mechanisms induced through selective IGF-1R targeting and off-target effects upon IR-B, it may be necessary to target both IGF ligands, insulin, IGF-1R, both hybrid receptor types, and IR-A in unison to have the greatest clinical impact while avoiding the unintended pharmacodynamic consequences. Finally, to the extent insulin can promote IR-A mediated oncogenic effects, one could hypothesize its use for the treatment of iatrogenic hyperglycemia should be avoided in patients harboring IGF-1R driven malignancies when other pharmacological options exist.

Of course a number of preclinical and clinical studies utilize a dual-targeting approach aimed at IGF-1R and extrinsic cascade-initiating RTKs or downstream mediators. Co-targeting c-kit and IGF-1R appears to be synergistic in EWS and small cell lung cancer (SCLC) cells [114]. A novel small molecule inhibitor of the IGF-1R/IR/ALK triad, GSK1838705A, has shown antitumor activity in human tumor models and should help elucidate the relationship of IGF-1R pathway activation in ALK-positive tumors noted within subtypes of NSCLC, lymphoma, and sarcoma [115]. And several phase I/II trials investigating mTOR/IGF-1R co-targeting have just been completed; everolimus/figitumumab [116] and cixutumumab/temsirolimus (Naing, personal communication), and ganitumab/rapamycin is on the horizon [117].

Certainly with respect to cancers of the lung, prostate and colon, which rely in part upon EGFR signaling for tumor growth and survival, dual targeting of IGF-1R and EGFR has garnered much interest, given the fact that reciprocal inhibition of one RTK in epithelial cancers often enhances expression of the other. Bispecific antibodies capable of binding both IGF-1R and EGFR are undergoing investigation [118] and numerous studies have combined IGF-1R targeted therapies with others against EGFR [118-121]. Such RTK crosstalk has also been observed for the human EGF receptor 2 (HER2), the target of trastuzumab in breast cancer, and preclinical studies indicate synergy with dual IGF-1R/HER2 targeting [122,123]. Finally, significant crosstalk between IGF-1R and the androgen receptor in prostate cancers [50,124] or estrogen receptor in breast cancers [125-129] has been observed, though this combination remains to be validated clinically. Of course, many preclinical studies, and some clinical ones, have assessed the role of IGF-1R antagonists in combination with traditional cytotoxic chemotherapy. However, this topic is beyond the scope of this review.

## 6. Conclusions

After a flourish of clinical trials designed to investigate the role of single-agent IGF-1R targeted therapy, much of the initial optimism has been tempered by the realization that only limited subsets of patients respond and, when they occur, such responses too often are unsustained beyond a few months. Not surprisingly, without significant response rates observed for common cancers (*i.e.*, breast, colon, lung, or prostate cancer), many pharmaceutical companies have ceased, or at a minimum delayed, clinical development of their respective IGF-1R inhibitors.

Though this deceleration in clinical trial implementation will assuredly limit patient access to IGF-1R targeted therapies in the short term, over the longer term it may actually serve a benefit by allowing the necessary preclinical science to be mature before committing a substantial number of patients to empiric, lengthy, and potentially suboptimal treatment. Drawing upon the lesions learned from other biologically targeted therapies such as trastuzumab, a number of questions must be answered if we are to make significant strides forward. Among just a few are as follows: (a) what predictive biomarkers allow for effective patient enrichment for those most likely to benefit; (b) what pharmacodynamic effects are associated with tumor control, and finally; (c) how should combinatorial therapies be advanced to avoid acquired resistance and maximize response duration. As the scientific community races to find answers, one anticipates in the not too distant future that IGF-1R antagonists will prove an essential weapon in the oncologist's arsenal to be wielded in unison with other biologically targeted agents.

## Figures and Tables

**Table 1.** Mechanisms of resistance to IGF-1R-targeted therapy.

**Pathway/Biomarker**	**Cancer Type**						**Effect**	**Ongoing Combination**	**Results**		
	**Breast**	**Lung**	**Colorectal**	**Pancreas**	**Prostate**	**Sarcoma**			***In vitro***	**Xenograft**	**Clinical**
**IR**	[130-132]		[130]		[130]		Crosstalk between IGF-1R and IR can provide signaling to IGF-1R cascade.	Yes	[132,133]	[132,133]	
**IGFBPs**	[134]	[135]	[135]				Interferes in interaction between IGF ligands and receptor.	No	[134,135]		
**HER-2**	[44,136]			[70,137,138]	[43,50,138]	[130]	Crosstalk signaling and alternative pathway	Yes	[44-47, 139]	[137,140]	[44,139]
**HSP90**				[130,141, 142]		[95,142]	Stabilizing IGF-1R and downstream effector proteins	Yes	[142]	[142]	
**EGFR**		[114]	[49,50,114, 120]	[107-109, 114]	[37,44,114]		Crosstalk signaling and alternative pathway	Yes	[45,48,52, 55,143]	[48,50,52, 55,143]	[144]
**mTOR**					[34,114, 124]		Safe and well tolerated with no unexpected toxicities	Yes	[27,143, 145]	[27,143, 145]	[116]
**FAK**				[106]			Interaction of FAK and IGF-1R through theirs N-terminal domains	No	[146-149]		

**Table 2.** Dual Targeting of IGF-1R and other pathways.

**Impaired Pathways/Biomarker**	**Compound**	**Potentia l Target**	**Potential Drug Type**	**Cancer Type**	**References**

**EGFR**		IGF-1R	MAb/TKI	Colorectal Cancer	[55,56,143]
	panitumumab	IGF-1R	MAb/TKI	Pancreatic Cancer	[70,137,138]
		IGF-1R	MAb/TKI	Colorectal Cancer	[55,56,143]
	cetuximab	IGF-1R	MAb	Pancreatic Cancer	[70,137,138,143]
		IGF-1R	MAb	Lung cancer	[143]
	erlotinib	IGF-1R	MAb	Pancreatic Cancer	[70,137,138]
		IGF-1R	MAb	Breast Cancer	[43,45,48,52,90,131,143,150-153]
	gefitinib	IGF-1R	TKI	Prostate cancer	[43,50,143]
		IGF-1R	TKI	Colorectal Cancer	[154]

**HER-2**	trastuzumab	IGF-1R	TKI	Breast Cancer	[44-47,90,131,139,143,150,151]
	pertuzumab	IGF-1R	MAb/TKI	Breast Cancer	[44,45,90,131,139,143,150,151]

**mTOR**	rapamycin	IGF-1R	MAb	Breast Cancer	[27,143,145,155,156]
	temsirolimus	IGF-1R	MAb	Breast Cancer	[27,143,145,155,156]
	temsirolimus	IGF-1R	MAb	Prostate cancer	[40,143,157]

**FAK**	FAK-siRNA	IGF-1R	MAb/TKI	Pancreatic Cancer	[147-149]

## References

[1] Salmon W.D., Daughaday W.H. (1957). A hormonally controlled serum factor which stimulates sulfate incorporation by cartilage *in vitro*. J. Lab. Clin. Med..

[2] Atzori F., Traina T.A., Ionta M.T., Massidda B. (2009). Targeting insulin-like growth factor type 1 receptor in cancer therapy. Target Oncol..

[3] Bahr C., Groner B. (2004). The insulin like growth factor-1 receptor (IGF-1R) as a drug target: Novel approaches to cancer therapy. Growth Horm. IGF Res..

[4] Bahr C., Groner B. (2005). The IGF-1 receptor and its contributions to metastatic tumor growth-novel approaches to the inhibition of IGF-1R function. Growth Factors.

[5] Camidge D.R., Dziadziuszko R., Hirsch F.R. (2009). The rationale and development of therapeutic insulin-like growth factor axis inhibition for lung and other cancers. Clin. Lung Cancer.

[6] Maki R.G. (2010). Small is beautiful: Insulin-like growth factors and their role in growth, development, and cancer. J. Clin. Oncol..

[7] Rosenzweig S.A., Atreya H.S. (2010). Defining the pathway to insulin-like growth factor system targeting in cancer. Biochem. Pharmacol..

[8] Moller D.E., Yokota A., Caro J.F., Flier J.S. (1989). Tissue-specific expression of two alternatively spliced insulin receptor mRNAs in man. Mol. Endocrinol..

[9] Ullrich A., Gray A., Tam A.W., Yang-Feng T., Tsubokawa M., Collins C., Henzel W., Le Bon T., Kathuria S., Chen E. (1986). Insulin-like growth factor I receptor primary structure: Comparison with insulin receptor suggests structural determinants that define functional specificity. EMBO J..

[10] Scotlandi K., Benini S., Sarti M., Serra M., Lollini P.L., Maurici D., Picci P., Manara M.C., Baldini N. (1996). Insulin-like growth factor I receptor-mediated circuit in Ewing's sarcoma/peripheral neuroectodermal tumor: A possible therapeutic target. Cancer Res..

[11] Baserga R. (2004). Targeting the IGF-1 receptor: From rags to riches. Eur. J. Cancer.

[12] Hubbard S.R., Wei L., Ellis L., Hendrickson W.A. (1994). Crystal structure of the tyrosine kinase domain of the human insulin receptor. Nature.

[13] Favelyukis S., Till J.H., Hubbard S.R., Miller W.T. (2001). Structure and autoregulation of the insulin-like growth factor 1 receptor kinase. Nat. Struct. Biol..

[14] Pautsch A., Zoephel A., Ahorn H., Spevak W., Hauptmann R., Nar H. (2001). Crystal structure of bisphosphorylated IGF-1 receptor kinase: Insight into domain movements upon kinase activation. Structure.

[15] Kaushansky A., Gordus A., Chang B., Rush J., MacBeath G. (2008). A quantitative study of the recruitment potential of all intracellular tyrosine residues on EGFR, FGFR1 and IGF1R. Mol. Biosyst..

[16] Heldin C.H. (1995). Dimerization of cell surface receptors in signal transduction. Cell.

[17] Baserga R., Peruzzi F., Reiss K. (2003). The IGF-1 receptor in cancer biology. Int. J. Cancer.

[18] De Meyts P., Palsgaard J., Sajid W., Theede A.M., Aladdin H. (2004). Structural biology of insulin and IGF-1 receptors. Novartis Found. Symp..

[19] Nakae J., Kido Y., Accili D. (2001). Distinct and overlapping functions of insulin and IGF-I receptors. Endocr. Rev..

[20] Pollak M. (2008). Insulin and insulin-like growth factor signalling in neoplasia. Nat. Rev. Cancer..

[21] Almeida A., Muleris M., Dutrillaux B., Malfoy B. (1994). The insulin-like growth factor I receptor gene is the target for the 15q26 amplicon in breast cancer. Genes Chromosomes Cancer.

[22] Tarn C., Rink L., Merkel E., Flieder D., Pathak H., Koumbi D., Testa J.R., Eisenberg B., von Mehren M., Godwin A.K. (2008). Insulin-like growth factor 1 receptor is a potential therapeutic target for gastrointestinal stromal tumors. Proc. Natl. Acad. Sci. USA.

[23] Armengol G., Knuutila S., Lluis F., Capella G., Miro R., Caballin M.R. (2000). DNA copy number changes and evaluation of MYC, IGF1R, and FES amplification in xenografts of pancreatic adenocarcinoma. Cancer Genet. Cytogenet..

[24] Natrajan R., Reis-Filho J.S., Little S.E., Messahel B., Brundler M.A., Dome J.S., Grundy P.E., Vujanic G.M., Pritchard-Jones K., Jones C. (2006). Blastemal expression of type I insulin-like growth factor receptor in Wilms' tumors is driven by increased copy number and correlates with relapse. Cancer Res..

[25] Nair P.N., De Armond D.T., Adamo M.L., Strodel W.E., Freeman J.W. (2001). Aberrant expression and activation of insulin-like growth factor-1 receptor (IGF-1R) are mediated by an induction of IGF-1R promoter activity and stabilization of IGF-1R mRNA and contributes to growth factor independence and increased survival of the pancreatic cancer cell line MIA PaCa-2. Oncogene.

[26] Benini S., Manara M.C., Cerisano V., Perdichizzi S., Strammiello R., Serra M., Picci P., Scotlandi K. (2004). Contribution of MEK/MAPK and PI3-K signaling pathway to the malignant behavior of Ewing's sarcoma cells: therapeutic prospects. Int. J. Cancer.

[27] Tao Y., Pinzi V., Bourhis J., Deutsch E. (2007). Mechanisms of disease: signaling of the insulin-like growth factor 1 receptor pathway--therapeutic perspectives in cancer. Nat. Clin. Pract. Oncol..

[28] Wan X., Helman L.J. (2007). The biology behind mTOR inhibition in sarcoma. Oncologist.

[29] Fasolo A., Sessa C. (2008). mTOR inhibitors in the treatment of cancer. Expert. Opin. Investig. Drugs.

[30] Chang Q., Li Y., White M.F., Fletcher J.A., Xiao S. (2002). Constitutive activation of insulin receptor substrate 1 is a frequent event in human tumors: Therapeutic implications. Cancer Res..

[31] Alberobello A.T., D'Esposito V., Marasco D., Doti N., Ruvo M., Bianco R., Tortora G., Esposito I., Fiory F., Miele C., Beguinot F., Formisano P. (2010). Selective disruption of insulin-like growth factor-1 (IGF-1) signaling via phosphoinositide-dependent kinase-1 prevents the protective effect of IGF-1 on human cancer cell death. J. Biol. Chem..

[32] Hirschfeld S., Helman L. (1994). Diverse roles of insulin-like growth factors in pediatric solid tumors. In Vivo.

[33] Kim S.Y., Wan X., Helman L.J. (2009). Targeting IGF-1R in the treatment of sarcomas: Past, present and future. Bull. Cancer.

[34] Dricu A., Kanter L., Wang M., Nilsson G., Hjertman M., Wejde J., Larsson O. (1999). Expression of the insulin-like growth factor 1 receptor (IGF-1R) in breast cancer cells: evidence for a regulatory role of dolichyl phosphate in the transition from an intracellular to an extracellular IGF-1 pathway. Glycobiology.

[35] Yu H., Berkel H. (1999). Insulin-like growth factors and cancer. J. La. State. Med. Soc..

[36] Moore M.G., Wetterau L.A., Francis M.J., Peehl D.M., Cohen P. (2003). Novel stimulatory role for insulin-like growth factor binding protein-2 in prostate cancer cells. Int. J. Cancer..

[37] Ma Z., Dong A., Kong M., Qian J. (2007). Silencing of the type 1 insulin-like growth factor receptor increases the sensitivity to apoptosis and inhibits invasion in human lung adenocarcinoma A549 cells. Cell Mol. Biol. Lett..

[38] Shevah O., Laron Z. (2007). Patients with congenital deficiency of IGF-I seem protected from the development of malignancies: a preliminary report. Growth Horm. IGF Res..

[39] Steuerman R., Shevah O., Laron Z. (2011). Congenital IGF1 deficiency tends to confer protection against post-natal development of malignancies. Eur. J. Endocrinol..

[40] Li R., Pourpak A., Morris S.W. (2009). Inhibition of the insulin-like growth factor-1 receptor (IGF1R) tyrosine kinase as a novel cancer therapy approach. J. Med. Chem..

[41] Frasca F., Pandini G., Vigneri R., Goldfine I.D. (2003). Insulin and hybrid insulin/IGF receptors are major regulators of breast cancer cells. Breast Dis..

[42] Vaira V., Lee C.W., Goel H.L., Bosari S., Languino L.R., Altieri D.C. (2007). Regulation of survivin expression by IGF-1/mTOR signaling. Oncogene.

[43] Jones H.E., Goddard L., Gee J.M., Hiscox S., Rubini M., Barrow D., Knowlden J.M., Williams S., Wakeling A.E., Nicholson R.I. (2004). Insulin-like growth factor-I receptor signalling and acquired resistance to gefitinib (ZD1839; Iressa) in human breast and prostate cancer cells. Endocr. Relat. Cancer.

[44] Lu Y., Zi X., Zhao Y., Mascarenhas D., Pollak M. (2001). Insulin-like growth factor-I receptor signaling and resistance to trastuzumab (Herceptin). J. Natl. Cancer Inst..

[45] Nahta R., Yuan L.X., Zhang B., Kobayashi R., Esteva F.J. (2005). Insulin-like growth factor-I receptor/human epidermal growth factor receptor 2 heterodimerization contributes to trastuzumab resistance of breast cancer cells. Cancer Res..

[46] Chakraborty A.K., Liang K., DiGiovanna M.P. (2008). Co-targeting insulin-like growth factor I receptor and HER2: Dramatic effects of HER2 inhibitors on nonoverexpressing breast cancer. Cancer Res..

[47] Browne B.C., Crown J., Venkatesan N., Duffy M.J., Clynes M., Slamon D., O'Donovan N. (2011). Inhibition of IGF1R activity enhances response to trastuzumab in HER-2-positive breast cancer cells. Ann. Oncol..

[48] van der Veeken J., Oliveira S., Schiffelers R.M., Storm G., van Bergen En Henegouwen P.M., Roovers R.C. (2009). Crosstalk between epidermal growth factor receptor- and insulin-like growth factor-1 receptor signaling: implications for cancer therapy. Curr. Cancer Drug Targets.

[49] Roznovanu S.L., Amalinci C., Radulescu D. (2005). Molecular mechanisms in hormone-resistant prostate cancer. Rev. Med. Chir. Soc. Med. Nat. Iasi..

[50] Wu J.D., Odman A., Higgins L.M., Haugk K., Vessella R., Ludwig D.L., Plymate S.R. (2005). *In vivo* effects of the human type I insulin-like growth factor receptor antibody A12 on androgen-dependent and androgen-independent xenograft human prostate tumors. Clin. Cancer Res..

[51] Kimura K., Markowski M., Bowen C., Gelmann E.P. (2001). Androgen blocks apoptosis of hormone-dependent prostate cancer cells. Cancer Res..

[52] Morgillo F., Woo J.K., Kim E.S., Hong W.K., Lee H.Y. (2006). Heterodimerization of insulin-like growth factor receptor/epidermal growth factor receptor and induction of survivin expression counteract the antitumor action of erlotinib. Cancer Res..

[53] Goetsch L., Gonzalez A., Leger O., Beck A., Pauwels P.J., Haeuw J.F., Corvaia N. (2005). A recombinant humanized anti-insulin-like growth factor receptor type I antibody (h7C10) enhances the antitumor activity of vinorelbine and anti-epidermal growth factor receptor therapy against human cancer xenografts. Int. J. Cancer.

[54] Gong Y., Yao E., Shen R., Goel A., Arcila M., Teruya-Feldstein J., Zakowski M.F., Frankel S., Peifer M., Thomas R.K., Ladanyi M., Pao W. (2009). High expression levels of total IGF-1R and sensitivity of NSCLC cells *in vitro* to an anti-IGF-1R antibody (R1507). PLoS One.

[55] Lu D., Zhang H., Koo H., Tonra J., Balderes P., Prewett M., Corcoran E., Mangalampalli V., Bassi R., Anselma D., Patel D., Kang X., Ludwig D.L., Hicklin D.J., Bohlen P., Witte L., Zhu Z. (2005). A fully human recombinant IgG-like bispecific antibody to both the epidermal growth factor receptor and the insulin-like growth factor receptor for enhanced antitumor activity. J. Biol. Chem..

[56] Bardelli A., Siena S. (2010). Molecular mechanisms of resistance to cetuximab and panitumumab in colorectal cancer. J. Clin. Oncol..

[57] Takahari D., Yamada Y., Okita N.T., Honda T., Hirashima Y., Matsubara J., Takashima A., Kato K., Hamaguchi T., Shirao K., Shimada Y., Shimoda T. (2009). Relationships of insulin-like growth factor-1 receptor and epidermal growth factor receptor expression to clinical outcomes in patients with colorectal cancer. Oncology.

[58] Hopfner M., Sutter A.P., Huether A., Baradari V., Scherubl H. (2006). Tyrosine kinase of insulin-like growth factor receptor as target for novel treatment and prevention strategies of colorectal cancer. World J. Gastroenterol..

[59] Donovan E.A., Kummar S. (2008). Role of insulin-like growth factor-1R system in colorectal carcinogenesis. Crit. Rev. Oncol. Hematol..

[60] Ma J., Pollak M.N., Giovannucci E., Chan J.M., Tao Y., Hennekens C.H., Stampfer M.J. (1999). Prospective study of colorectal cancer risk in men and plasma levels of insulin-like growth factor (IGF)-I and IGF-binding protein-3. J. Natl. Cancer Inst..

[61] Ma J., Stampfer M., Pollak M. (2000). RESPONSE: More about: Prospective study of colorectal cancer risk in men and plasma levels of insulin-like growth factor (IGF)-I and IGF- binding protein-3. J. Natl. Cancer Inst..

[62] Reidy D.L., Vakiani E., Fakih M.G., Saif M.W., Hecht J.R., Goodman-Davis N., Hollywood E., Shia J., Schwartz J., Chandrawansa K., Dontabhaktuni A., Youssoufian H., Solit D.B., Saltz L.B. (2010). Randomized, phase II study of the insulin-like growth factor-1 receptor inhibitor IMC-A12, with or without cetuximab, in patients with cetuximab- or panitumumab-refractory metastatic colorectal cancer. J. Clin. Oncol..

[63] Hellawell G.O., Turner G.D., Davies D.R., Poulsom R., Brewster S.F., Macaulay V.M. (2002). Expression of the type 1 insulin-like growth factor receptor is up-regulated in primary prostate cancer and commonly persists in metastatic disease. Cancer Res..

[64] Hakam A., Fang Q., Karl R., Coppola D. (2003). Coexpression of IGF-1R and c-Src proteins in human pancreatic ductal adenocarcinoma. Dig. Dis. Sci..

[65] Freeman J.W., Mattingly C.A., Strodel W.E. (1995). Increased tumorigenicity in the human pancreatic cell line MIA PaCa-2 is associated with an aberrant regulation of an IGF-1 autocrine loop and lack of expression of the TGF-beta type RII receptor. J. Cell. Physiol..

[66] Bao B., Wang Z., Li Y., Kong D., Ali S., Banerjee S., Ahmad A., Sarkar F.H. (2011). The complexities of obesity and diabetes with the development and progression of pancreatic cancer. Biochim. Biophys. Acta.

[67] Tomizawa M., Shinozaki F., Sugiyama T., Yamamoto S., Sueishi M., Yoshida T. (2010). Insulin-like growth factor-I receptor in proliferation and motility of pancreatic cancer. World. J. Gastroenterol..

[68] Bergmann U., Funatomi H., Yokoyama M., Beger H.G., Korc M. (1995). Insulin-like growth factor I overexpression in human pancreatic cancer: evidence for autocrine and paracrine roles. Cancer Res..

[69] Stoeltzing O., Liu W., Reinmuth N., Fan F., Parikh A.A., Bucana C.D., Evans D.B., Semenza G.L., Ellis L.M. (2003). Regulation of hypoxia-inducible factor-1alpha, vascular endothelial growth factor, and angiogenesis by an insulin-like growth factor-I receptor autocrine loop in human pancreatic cancer. Am. J. Pathol..

[70] Maloney E.K., McLaughlin J.L., Dagdigian N.E., Garrett L.M., Connors K.M., Zhou X.M., Blattler W.A., Chittenden T., Singh R. (2003). An anti-insulin-like growth factor I receptor antibody that is a potent inhibitor of cancer cell proliferation. Cancer Res..

[71] Kim W.Y., Jin Q., Oh S.H., Kim E.S., Yang Y.J., Lee D.H., Feng L., Behrens C., Prudkin L., Miller Y.E., Lee J.J., Lippman S.M., Hong W.K., Wistuba II, Lee H.Y. (2009). Elevated epithelial insulin-like growth factor expression is a risk factor for lung cancer development. Cancer Res..

[72] Dziadziuszko R., Camidge D.R., Hirsch F.R. (2008). The insulin-like growth factor pathway in lung cancer. J. Thorac. Oncol..

[73] Yu H., Spitz M.R., Mistry J., Gu J., Hong W.K., Wu X. (1999). Plasma levels of insulin-like growth factor-I and lung cancer risk: A case-control analysis. J. Natl. Cancer Inst..

[74] London S.J., Yuan J.M., Travlos G.S., Gao Y.T., Wilson R.E., Ross R.K., Yu M.C. (2002). Insulin-like growth factor I, IGF-binding protein 3, and lung cancer risk in a prospective study of men in China. J. Natl. Cancer Inst..

[75] Renehan A.G., Zwahlen M., Minder C., O'Dwyer S.T., Shalet S.M., Egger M. (2004). Insulin-like growth factor (IGF)-I, IGF binding protein-3, and cancer risk: Systematic review and meta-regression analysis. Lancet.

[76] Gualberto A., Karp D.D. (2009). Development of the monoclonal antibody figitumumab, targeting the insulin-like growth factor-1 receptor, for the treatment of patients with non-small-cell lung cancer. Clin. Lung Cancer.

[77] Karp D.D., Paz-Ares L.G., Novello S., Haluska P., Garland L., Cardenal F., Blakely L.J., Eisenberg P.D., Langer C.J., Blumenschein G., Johnson F.M., Green S., Gualberto A. (2009). Phase II study of the anti-insulin-like growth factor type 1 receptor antibody CP-751,871 in combination with paclitaxel and carboplatin in previously untreated, locally advanced, or metastatic non-small-cell lung cancer. J. Clin. Oncol..

[78] Méndez M., Custodio A., Provencio M. (2011). New molecular targeted therapies for advanced non-small-cell lung cancer. J. Thoracic Dis..

[79] Gualberto A., Dolled-Filhart M., Gustavson M., Christiansen J.J., Wang Y.F., Hixon M.L., Reynolds J.M., McDonald S., Ang A., Rimm D.L., Langer C., Blakely J., Garland L.L., Paz-Ares L., Karp D.D., Lee A.V. (2010). Molecular Analysis of Non-Small Cell Lung Cancer (NSCLC) Identifies Subsets with Different Sensitivity to Insulin like Growth Factor I Receptor (IGF-IR) Inhibition. Clin. Cancer Res..

[80] Toretsky J.A., Kalebic T., Blakesley V., LeRoith D., Helman L.J. (1997). The insulin-like growth factor-I receptor is required for EWS/FLI-1 transformation of fibroblasts. J. Biol. Chem..

[81] Cironi L., Riggi N., Provero P., Wolf N., Suva M.L., Suva D., Kindler V., Stamenkovic I. (2008). IGF1 is a common target gene of Ewing's sarcoma fusion proteins in mesenchymal progenitor cells. PLoS. ONE.

[82] Prieur A., Tirode F., Cohen P., Delattre O. (2004). EWS/FLI-1 silencing and gene profiling of Ewing cells reveal downstream oncogenic pathways and a crucial role for repression of insulin-like growth factor binding protein 3. Mol. Cell Biol..

[83] Toretsky J.A., Steinberg S.M., Thakar M., Counts D., Pironis B., Parente C., Eskenazi A., Helman L., Wexler L.H. (2001). Insulin-like growth factor type 1 (IGF-1) and IGF binding protein-3 in patients with Ewing sarcoma family of tumors. Cancer.

[84] Windsor R., Strauss S., Seddon B., Whelan J. (2009). Experimental therapies in Ewing's sarcoma. Expert. Opin. Investig. Drugs.

[85] Yuen J.S., Macaulay V.M. (2008). Targeting the type 1 insulin-like growth factor receptor as a treatment for cancer. Expert. Opin. Ther. Targets.

[86] Janeway K.A., Zhu M.J., Barretina J., Perez-Atayde A., Demetri G.D., Fletcher J.A. (2010). Strong expression of IGF1R in pediatric gastrointestinal stromal tumors without IGF1R genomic amplification. Int. J. Cancer.

[87] Pandini G., Wurch T., Akla B., Corvaia N., Belfiore A., Goetsch L. (2007). Functional responses and *in vivo* anti-tumour activity of h7C10: a humanised monoclonal antibody with neutralising activity against the insulin-like growth factor-1 (IGF-1) receptor and insulin/IGF-1 hybrid receptors. Eur. J. Cancer.

[88] Buck E., Mulvihill M. (2011). Small molecule inhibitors of the IGF-1R/IR axis for the treatment of cancer. Expert. Opin. Investig. Drugs.

[89] Hixon M.L., Paccagnella L., Millham R., Perez-Olle R., Gualberto A. (2010). Development of inhibitors of the IGF-IR/PI3K/Akt/mTOR pathway. Rev. Recent Clin. Trials.

[90] Hartog H., Wesseling J., Boezen H.M., van der Graaf W.T. (2007). The insulin-like growth factor 1 receptor in cancer: Old focus, new future. Eur. J. Cancer.

[91] Olmos D., Martins A.S., Jones R.L., Alam S., Scurr M., Judson I.R. (2011). Targeting the Insulin-Like Growth Factor 1 Receptor in Ewing's Sarcoma: Reality and Expectations. Sarcoma.

[92] Wolpin B.M., Meyerhardt J.A., Chan A.T., Ng K., Chan J.A., Wu K., Pollak M.N., Giovannucci E.L., Fuchs C.S. (2009). Insulin, the insulin-like growth factor axis, and mortality in patients with nonmetastatic colorectal cancer. J. Clin. Oncol..

[93] Merchant M.S., Woo C.W., Mackall C.L., Thiele C.J. (2002). Potential use of imatinib in Ewing's Sarcoma: evidence for *in vitro* and *in vivo* activity. J. Natl. Cancer Inst..

[94] Scotlandi K., Manara M.C., Hattinger C.M., Benini S., Perdichizzi S., Pasello M., Bacci G., Zanella L., Bertoni F., Picci P., Serra M. (2005). Prognostic and therapeutic relevance of HER2 expression in osteosarcoma and Ewing's sarcoma. Eur. J. Cancer.

[95] Martins A.S., Ordonez J.L., Garcia-Sanchez A., Herrero D., Sevillano V., Osuna D., Mackintosh C., Caballero G., Otero A.P., Poremba C., Madoz-Gurpide J., de Alava E. (2008). A pivotal role for heat shock protein 90 in Ewing sarcoma resistance to anti-insulin-like growth factor 1 receptor treatment: *in vitro* and *in vivo* study. Cancer Res..

[96] Bond M., Bernstein M.L., Pappo A., Schultz K.R., Krailo M., Blaney S.M., Adamson P.C. (2008). A phase II study of imatinib mesylate in children with refractory or relapsed solid tumors: A Children's Oncology Group study. Pediatr. Blood Cancer.

[97] Timeus F., Crescenzio N., Fandi A., Doria A., Foglia L., Cordero di Montezemolo L. (2008). *In vitro* antiproliferative and antimigratory activity of dasatinib in neuroblastoma and Ewing sarcoma cell lines. Oncol. Rep..

[98] Martins A.S., Mackintosh C., Martin D.H., Campos M., Hernandez T., Ordonez J.L., de Alava E. (2006). Insulin-like growth factor I receptor pathway inhibition by ADW742, alone or in combination with imatinib, doxorubicin, or vincristine, is a novel therapeutic approach in Ewing tumor. Clin. Cancer Res..

[99] Andersson M.K., Aman P. (2008). Proliferation of Ewing sarcoma cell lines is suppressed by the receptor tyrosine kinase inhibitors gefitinib and vandetanib. Cancer Cell. Int..

[100] Potratz J.C., Saunders D.N., Wai D.H., Ng T.L., McKinney S.E., Carboni J.M., Gottardis M.M., Triche T.J., Jurgens H., Pollak M.N., Aparicio S.A., Sorensen P.H. (2010). Synthetic lethality screens reveal RPS6 and MST1R as modifiers of insulin-like growth factor-1 receptor inhibitor activity in childhood sarcomas. Cancer Res..

[101] Huang F., Greer A., Hurlburt W., Han X., Hafezi R., Wittenberg G.M., Reeves K., Chen J., Robinson D., Li A., Lee F.Y., Gottardis M.M., Clark E., Helman L., Attar R.M., Dongre A., Carboni J.M. (2009). The mechanisms of differential sensitivity to an insulin-like growth factor-1 receptor inhibitor (BMS-536924) and rationale for combining with EGFR/HER2 inhibitors. Cancer Res..

[102] Girnita L., Girnita A., Larsson O. (2003). Mdm2-dependent ubiquitination and degradation of the insulin-like growth factor 1 receptor. Proc. Natl. Acad. Sci. USA.

[103] Froment P., Dupont J., Christophe-Marine J. (2008). Mdm2 exerts pro-apoptotic activities by antagonizing insulin-like growth factor-I-mediated survival. Cell Cycle.

[104] Kovar H., Auinger A., Jug G., Aryee D., Zoubek A., Salzer-Kuntschik M., Gadner H. (1993). Narrow spectrum of infrequent p53 mutations and absence of MDM2 amplification in Ewing tumours. Oncogene.

[105] Cao L., Yu Y., Darko I., Currier D., Mayeenuddin L.H., Wan X., Khanna C., Helman L.J. (2008). Addiction to elevated insulin-like growth factor I receptor and initial modulation of the AKT pathway define the responsiveness of rhabdomyosarcoma to the targeting antibody. Cancer Res..

[106] Ludwig J.A., Weinstein J.N. (2005). Biomarkers in cancer staging, prognosis and treatment selection. Nat. Rev. Cancer.

[107] Garofalo C., Manara M.C., Nicoletti G., Marino M.T., Lollini P.L., Astolfi A., Pandini G., Lopez-Guerrero J.A., Schaefer K.L., Belfiore A., Picci P., Scotlandi K. (2011). Efficacy of and resistance to anti-IGF-1R therapies in Ewing's sarcoma is dependent on insulin receptor signaling. Oncogene.

[108] Kolb E.A., Gorlick R., Houghton P.J., Morton C.L., Lock R., Carol H., Reynolds C.P., Maris J.M., Keir S.T., Billups C.A., Smith M.A. (2008). Initial testing (stage 1) of a monoclonal antibody (SCH 717454) against the IGF-1 receptor by the pediatric preclinical testing program. Pediatr. Blood Cancer.

[109] Mulvihill M.J., Cooke A., Rosenfeld-Franklin M., Buck E., Foreman K., Landfair D., O'Connor M., Pirritt C., Sun Y., Yao Y., Arnold L.D., Gibson N.W., Ji Q.S. (2009). Discovery of OSI-906: A selective and orally efficacious dual inhibitor of the IGF-1 receptor and insulin receptor. Future Med. Chem..

[110] McKinley E.T., Bugaj J.E., Zhao P., Guleryuz S., Mantis C., Gokhale P.C., Wild R., Manning H.C. (2011). 18FDG-PET predicts pharmacodynamic response to OSI-906, a dual IGF-1R/IR inhibitor, in preclinical mouse models of lung cancer. Clin. Cancer Res..

[111] Buck E., Gokhale P.C., Koujak S., Brown E., Eyzaguirre A., Tao N., Rosenfeld-Franklin M., Lerner L., Chiu M.I., Wild R., Epstein D., Pachter J.A., Miglarese M.R. (2010). Compensatory insulin receptor (IR) activation on inhibition of insulin-like growth factor-1 receptor (IGF-1R): rationale for cotargeting IGF-1R and IR in cancer. Mol. Cancer Ther..

[112] Dong J., Demarest S.J., Sereno A., Tamraz S., Langley E., Doern A., Snipas T., Perron K., Joseph I., Glaser S.M., Ho S.N., Reff M.E., Hariharan K. (2010). Combination of two insulin-like growth factor-I receptor inhibitory antibodies targeting distinct epitopes leads to an enhanced antitumor response. Mol. Cancer Ther..

[113] Gao J., Chesebrough J.W., Cartlidge S.A., Ricketts S.A., Incognito L., Veldman-Jones M., Blakey D.C., Tabrizi M., Jallal B., Trail P.A., Coats S., Bosslet K., Chang Y.S. (2011). Dual IGF-I/II-neutralizing antibody MEDI-573 potently inhibits IGF signaling and tumor growth. Cancer Res..

[114] Camirand A., Pollak M. (2004). Co-targeting IGF-1R and c-kit: Synergistic inhibition of proliferation and induction of apoptosis in H 209 small cell lung cancer cells. Br. J. Cancer.

[115] Sabbatini P., Korenchuk S., Rowand J.L., Groy A., Liu Q., Leperi D., Atkins C., Dumble M., Yang J., Anderson K., Kruger R.G., Gontarek R.R., Maksimchuk K.R., Suravajjala S., Lapierre R.R., Shotwell J.B., Wilson J.W., Chamberlain S.D., Rabindran S.K., Kumar R. (2009). GSK1838705A inhibits the insulin-like growth factor-1 receptor and anaplastic lymphoma kinase and shows antitumor activity in experimental models of human cancers. Mol. Cancer Ther..

[116] Quek R., Wang Q., Morgan J.A., Shapiro G.I., Butrynski J.E., Ramaiya N., Huftalen T., Jederlinic N., Manola J., Wagner A.J., Demetri G.D., George S. (2011). Combination mTOR and IGF-1R inhibition: phase I trial of everolimus and figitumumab in patients with advanced sarcomas and other solid tumors. Clin. Cancer Res..

[117] Beltran P.J., Chung Y.A., Moody G., Mitchell P., Cajulis E., Vonderfecht S., Kendall R., Radinsky R., Calzone F.J. (2011). Efficacy of Ganitumab (AMG 479), Alone and in Combination With Rapamycin, in Ewing's and Osteogenic Sarcoma Models. J. Pharmacol. Exp. Ther..

[118] Dong J., Sereno A., Aivazian D., Langley E., Miller B., Snyder W., Chan E., Cantele M., Morena R., Joseph I., Boccia A., Virata C., Gamez J., Yco G., Favis M., Wu X., Graff C., Wang Q., Rohde E., Berquist L., Huang F., Zhang Y., Gao S., Ho S., Demarest S., Reff M., Hariharan K., Glaser S. (2011). A stable IgG-like bispecific antibody targeting the epidermal growth factor receptor and the type I insulin-like growth factor receptor demonstrates superior anti-tumor activity. MAbs.

[119] Nguyen K.S., Kobayashi S., Costa D.B. (2009). Acquired resistance to epidermal growth factor receptor tyrosine kinase inhibitors in non-small-cell lung cancers dependent on the epidermal growth factor receptor pathway. Clin. Lung Cancer.

[120] Buck E., Eyzaguirre A., Rosenfeld-Franklin M., Thomson S., Mulvihill M., Barr S., Brown E., O'Connor M., Yao Y., Pachter J., Miglarese M., Epstein D., Iwata K.K., Haley J.D., Gibson N.W., Ji Q.S. (2008). Feedback mechanisms promote cooperativity for small molecule inhibitors of epidermal and insulin-like growth factor receptors. Cancer Res..

[121] Guix M., Faber A.C., Wang S.E., Olivares M.G., Song Y., Qu S., Rinehart C., Seidel B., Yee D., Arteaga C.L., Engelman J.A. (2008). Acquired resistance to EGFR tyrosine kinase inhibitors in cancer cells is mediated by loss of IGF-binding proteins. J. Clin. Invest..

[122] Haluska P., Carboni J.M., TenEyck C., Attar R.M., Hou X., Yu C., Sagar M., Wong T.W., Gottardis M.M., Erlichman C. (2008). HER receptor signaling confers resistance to the insulin-like growth factor-I receptor inhibitor, BMS-536924. Mol. Cancer Ther..

[123] Wheeler D.L., Huang S., Kruser T.J., Nechrebecki M.M., Armstrong E.A., Benavente S., Gondi V., Hsu K.T., Harari P.M. (2008). Mechanisms of acquired resistance to cetuximab: Role of HER (ErbB) family members. Oncogene.

[124] Wu J.D., Haugk K., Woodke L., Nelson P., Coleman I., Plymate S.R. (2006). Interaction of IGF signaling and the androgen receptor in prostate cancer progression. J. Cell. Biochem..

[125] Chong K., Subramanian A., Sharma A., Mokbel K. (2011). Measuring IGF-1, ER-alpha and EGFR expression can predict tamoxifen-resistance in ER-positive breast cancer. Anticancer Res..

[126] Chong K.Y., Subramanian A., Mokbel K., Sharma A.K. (2011). The prognostic significance of the insulin-like growth factor-1 ligand and receptor expression in breast cancer tissue. J. Surg. Oncol..

[127] Song R.X., Barnes C.J., Zhang Z., Bao Y., Kumar R., Santen R.J. (2004). The role of Shc and insulin-like growth factor 1 receptor in mediating the translocation of estrogen receptor alpha to the plasma membrane. Proc. Natl. Acad. Sci. USA.

[128] Song R.X., McPherson R.A., Adam L., Bao Y., Shupnik M., Kumar R., Santen R.J. (2002). Linkage of rapid estrogen action to MAPK activation by ERalpha-Shc association and Shc pathway activation. Mol. Endocrinol..

[129] Song R.X., Santen R.J., Kumar R., Adam L., Jeng M.H., Masamura S., Yue W. (2002). Adaptive mechanisms induced by long-term estrogen deprivation in breast cancer cells. Mol. Cell. Endocrinol..

[130] Wahner Hendrickson A.E., Haluska P., Schneider P.A., Loegering D.A., Peterson K.L., Attar R., Smith B.D., Erlichman C., Gottardis M., Karp J.E., Carboni J.M., Kaufmann S.H. (2009). Expression of insulin receptor isoform A and insulin-like growth factor-1 receptor in human acute myelogenous leukemia: Effect of the dual-receptor inhibitor BMS-536924 *in vitro*. Cancer Res..

[131] Papa V., Gliozzo B., Clark G.M., McGuire W.L., Moore D., Fujita-Yamaguchi Y., Vigneri R., Goldfine I.D., Pezzino V. (1993). Insulin-like growth factor-I receptors are overexpressed and predict a low risk in human breast cancer. Cancer Res..

[132] Milazzo G., Giorgino F., Damante G., Sung C., Stampfer M.R., Vigneri R., Goldfine I.D., Belfiore A. (1992). Insulin receptor expression and function in human breast cancer cell lines. Cancer Res..

[133] Ulanet D.B., Ludwig D.L., Kahn C.R., Hanahan D. (2010). Insulin receptor functionally enhances multistage tumor progression and conveys intrinsic resistance to IGF-1R targeted therapy. Proc. Natl. Acad. Sci. USA.

[134] Jerome L., Alami N., Belanger S., Page V., Yu Q., Paterson J., Shiry L., Pegram M., Leyland-Jones B. (2006). Recombinant human insulin-like growth factor binding protein 3 inhibits growth of human epidermal growth factor receptor-2-overexpressing breast tumors and potentiates herceptin activity *in vivo*. Cancer Res..

[135] Alami N., Page V., Yu Q., Jerome L., Paterson J., Shiry L., Leyland-Jones B. (2008). Recombinant human insulin-like growth factor-binding protein 3 inhibits tumor growth and targets the Akt pathway in lung and colon cancer models. Growth Horm. IGF Res..

[136] Camirand A., Lu Y., Pollak M. (2002). Co-targeting HER2/ErbB2 and insulin-like growth factor-1 receptors causes synergistic inhibition of growth in HER2-overexpressing breast cancer cells. Med. Sci. Monit..

[137] Burtrum D., Zhu Z., Lu D., Anderson D.M., Prewett M., Pereira D.S., Bassi R., Abdullah R., Hooper A.T., Koo H., Jimenez X., Johnson D., Apblett R., Kussie P., Bohlen P., Witte L., Hicklin D.J., Ludwig D.L. (2003). A fully human monoclonal antibody to the insulin-like growth factor I receptor blocks ligand-dependent signaling and inhibits human tumor growth *in vivo*. Cancer Res..

[138] Tonra J.R., Deevi D.S., Corcoran E., Li H., Wang S., Carrick F.E., Hicklin D.J. (2006). Synergistic antitumor effects of combined epidermal growth factor receptor and vascular endothelial growth factor receptor-2 targeted therapy. Clin. Cancer Res..

[139] Balana M.E., Labriola L., Salatino M., Movsichoff F., Peters G., Charreau E.H., Elizalde P.V. (2001). Activation of ErbB-2 via a hierarchical interaction between ErbB-2 and type I insulin-like growth factor receptor in mammary tumor cells. Oncogene.

[140] Beltran P.J., Mitchell P., Chung Y.A., Cajulis E., Lu J., Belmontes B., Ho J., Tsai M.M., Zhu M., Vonderfecht S., Baserga R., Kendall R., Radinsky R., Calzone F.J. (2009). AMG 479, a fully human anti-insulin-like growth factor receptor type I monoclonal antibody, inhibits the growth and survival of pancreatic carcinoma cells. Mol. Cancer. Ther..

[141] LeRoith D., Roberts C.T. (2003). The insulin-like growth factor system and cancer. Cancer Lett..

[142] Lang S.A., Moser C., Gaumann A., Klein D., Glockzin G., Popp F.C., Dahlke M.H., Piso P., Schlitt H.J., Geissler E.K., Stoeltzing O. (2007). Targeting heat shock protein 90 in pancreatic cancer impairs insulin-like growth factor-I receptor signaling, disrupts an interleukin-6/signal-transducer and activator of transcription 3/hypoxia-inducible factor-1alpha autocrine loop, and reduces orthotopic tumor growth. Clin. Cancer Res..

[143] Hewish M., Chau I., Cunningham D. (2009). Insulin-like growth factor 1 receptor targeted therapeutics: novel compounds and novel treatment strategies for cancer medicine. Recent Pat. Anticancer Drug Discov..

[144] Heidegger I., Pircher A., Klocker H., Massoner P. (2011). Targeting the insulin-like growth factor network in cancer therapy. Cancer Biol. Ther..

[145] Haruta T., Uno T., Kawahara J., Takano A., Egawa K., Sharma P.M., Olefsky J.M., Kobayashi M. (2000). A rapamycin-sensitive pathway down-regulates insulin signaling via phosphorylation and proteasomal degradation of insulin receptor substrate-1. Mol. Endocrinol..

[146] Liu T.J., LaFortune T., Honda T., Ohmori O., Hatakeyama S., Meyer T., Jackson D., de Groot J., Yung W.K. (2007). Inhibition of both focal adhesion kinase and insulin-like growth factor-I receptor kinase suppresses glioma proliferation *in vitro* and *in vivo*. Mol. Cancer Ther..

[147] Liu W., Bloom D.A., Cance W.G., Kurenova E.V., Golubovskaya V.M., Hochwald S.N. (2008). FAK and IGF-IR interact to provide survival signals in human pancreatic adenocarcinoma cells. Carcinogenesis.

[148] Zheng D., Golubovskaya V., Kurenova E., Wood C., Massoll N.A., Ostrov D., Cance W.G., Hochwald S.N. (2010). A novel strategy to inhibit FAK and IGF-1R decreases growth of pancreatic cancer xenografts. Mol. Carcinog..

[149] Zheng D., Kurenova E., Ucar D., Golubovskaya V., Magis A., Ostrov D., Cance W.G., Hochwald S.N. (2009). Targeting of the protein interaction site between FAK and IGF-1R. Biochem. Biophys. Res. Commun..

[150] Bonneterre J., Peyrat J.P., Beuscart R., Demaille A. (1990). Prognostic significance of insulin-like growth factor 1 receptors in human breast cancer. Cancer Res..

[151] Nielsen T.O., Andrews H.N., Cheang M., Kucab J.E., Hsu F.D., Ragaz J., Gilks C.B., Makretsov N., Bajdik C.D., Brookes C., Neckers L.M., Evdokimova V., Huntsman D.G., Dunn S.E. (2004). Expression of the insulin-like growth factor I receptor and urokinase plasminogen activator in breast cancer is associated with poor survival: potential for intervention with 17-allylamino geldanamycin. Cancer Res..

[152] Agrawal A., Gutteridge E., Gee J.M., Nicholson R.I., Robertson J.F. (2005). Overview of tyrosine kinase inhibitors in clinical breast cancer. Endocr. Relat. Cancer.

[153] Jin Q., Esteva F.J. (2008). Cross-talk between the ErbB/HER family and the type I insulin-like growth factor receptor signaling pathway in breast cancer. J. Mammary Gland Biol. Neoplasia.

[154] Jones H.E., Gee J.M., Barrow D., Tonge D., Holloway B., Nicholson R.I. (2006). Inhibition of insulin receptor isoform-A signalling restores sensitivity to gefitinib in previously de novo resistant colon cancer cells. Br. J. Cancer.

[155] Kurmasheva R.T., Huang S., Houghton P.J. (2006). Predicted mechanisms of resistance to mTOR inhibitors. Br. J. Cancer.

[156] Wan X., Harkavy B., Shen N., Grohar P., Helman L.J. (2007). Rapamycin induces feedback activation of Akt signaling through an IGF-1R-dependent mechanism. Oncogene.

[157] Masiello D., Mohi M.G., McKnight N.C., Smith B., Neel B.G., Balk S.P., Bubley G.J. (2007). Combining an mTOR antagonist and receptor tyrosine kinase inhibitors for the treatment of prostate cancer. Cancer Biol. Ther..

